# Effects of a Cloud-Based Synchronous Telehealth Program on Valvular Regurgitation Regression: Retrospective Study

**DOI:** 10.2196/68929

**Published:** 2025-04-23

**Authors:** Li-Tan Yang, Chi-Han Wu, Jen-Kuang Lee, Wei-Jyun Wang, Ying-Hsien Chen, Ching-Chang Huang, Chi-Sheng Hung, Kuang-Chien Chiang, Yi-Lwun Ho, Hui-Wen Wu

**Affiliations:** 1 Division of Cardiology Department of Internal Medicine National Taiwan University Hospital Taipei Taiwan; 2 Department of Internal Medicine College of Medicine National Taiwan University Taipei Taiwan; 3 Telehealth Center National Taiwan University Hospital Taipei Taiwan

**Keywords:** mitral regurgitation, tricuspid regurgitation, telehealth, telemedicine, cardiac remodeling

## Abstract

**Background:**

Telemedicine has been associated with better cardiovascular outcomes, but its effects on the regression of mitral regurgitation (MR) and tricuspid regurgitation (TR) remain unknown.

**Objective:**

This study aimed to evaluate whether telemedicine could facilitate the regression of MR and TR compared to usual care and whether it was associated with better survival.

**Methods:**

This retrospective cohort study enrolled consecutive patients with moderate or greater MR or TR from 2010 through 2020, excluding those with concomitant aortic stenosis, aortic regurgitation, or mitral stenosis greater than mild severity. All patients underwent follow-up transthoracic echocardiography (TTE) at least 3 months apart. Patients receiving telehealth services for at least two weeks within 90 days of baseline TTE were categorized as the telehealth group; the remainder constituted the nontelehealth group. Telemedicine participants transmitted daily biometric data—blood pressure, pulse rate, blood glucose, electrocardiogram, and oxygen saturation—to a cloud-based platform for timely monitoring. Experienced case managers regularly contacted patients and initiated immediate action for concerning measurements. The primary endpoint was MR or TR regression from ≥moderate to <moderate. The secondary endpoint was all-cause death (ACD). The last follow-up ended in December 2022.

**Results:**

The MR cohorts consisted of 264 patients (mean age 67 years), including 97 regressors and 74 telehealth participants. Telehealth participation (hazard ratio 2.20, 95% CI 1.35-3.58; *P*=.001) was robustly associated with MR regression; MR regressors were linked to reverse cardiac remodeling, indicated by improved left ventricular ejection fraction (LVEF), and reduced left ventricular (LV) and left atrial (LA) dimensions (all *P*≤.005). Determinants of ACD were age (*P*<.001), LVEF (*P*<.001), percutaneous coronary intervention (*P*<.001), and MR regressors (*P*=.02). The TR cohort consisted of 245 patients (mean age 68 years), including 87 TR regressors and 61 telehealth participants. Telehealth (*P*=.05) was one of the univariable determinants of TR regression, while beta-blocker use (*P*=.048) and baseline TR severity (*P*=.01) remained strong predictors of TR regression in multivariable analysis.

**Conclusions:**

Patients in the telehealth group were 2.2 times more likely to experience MR regression. Moreover, MR regressors had better survival and reverse cardiac remodeling compared to nonregressors. These findings may have important implications for future guidelines.

## Introduction

Valvular heart disease (VHD), which poses a substantial medical burden and is reported to be underdiagnosed, has affected 11% of people aged more than 65 years old [1,2]. Of these, mitral regurgitation (MR) is one of the most common VHD in several population-based studies; it precipitates atrial fibrillation, left-sided heart failure (HF), and reduces life expectancy [3-6]. Tricuspid regurgitation (TR), another common VHD, often develops secondary to left-sided heart disease or pulmonary hypertension, which also increases the risk of all-cause death (ACD) [7,8]. Timely intervention before irreversible cardiac remodeling could prevent detrimental outcomes [9-11], highlighting the importance of early detection and close monitoring.

The management of MR is determined by its etiology. In primary MR, regular monitoring via transthoracic echocardiography (TTE) is essential. Surgery is recommended for severe MR with intolerable symptoms or in asymptomatic patients with left ventricular dysfunction [10]. Secondary MR, on the other hand, is managed with guideline-directed medical therapy, including angiotensin-converting enzyme inhibitors, angiotensin receptor blockers, beta-blockers, and aldosterone antagonists, to achieve left ventricular (LV) reverse remodeling [10]. In patients presenting with right-sided heart failure symptoms caused by TR, diuretics may offer symptomatic relief [10]. For both MR and TR, timely intervention before irreversible cardiac remodeling occurs can prevent adverse outcomes [9-11], emphasizing the need for early detection and close monitoring.

The demands for telemedicine have surged in the post–COVID-19 era, and a plethora of studies have demonstrated its benefits in reducing mortality and HF hospitalization for patients with chronic cardiovascular (CV) diseases [12-16]. Moreover, research on the integration of handheld ultrasound into telemedicine has been emerging across various medical disciplines, including obstetrics [17], trauma medicine [18], and pulmonology [19], facilitating clinical decision-making [20] and reducing medical costs [21].

However, the associations between telemonitoring and VHD remained largely unknown [22]. Previously, we were the first to report that patients receiving telehealth services, despite a higher burden of comorbidities, exhibited comparable rates of MR and TR progression from ≤mild-moderate to ≥moderate severity compared to the control group [23]. Nevertheless, whether telemonitoring can promote the regression of MR or TR from ≥moderate to <moderate remains uncertain.

In this context, our study aimed to (1) compare the profiles of regressors and nonregressors in MR and TR; (2) identify factors influencing MR/TR regression, including telemedicine versus standard care; and (3) assess the determinants of survival.

## Methods

### Study Population

We retrospectively enrolled patients admitted to the cardiology ward at National Taiwan University Hospital (NTUH) between 2010 and 2020. The inclusion criteria were as follows: (1) patients with at least two TTEs performed at least three months apart (eg, baseline and last TTEs); (2) baseline TTE indicating moderate, moderate-severe, or severe MR or TR; and (3) absence of moderate or greater aortic stenosis, aortic regurgitation, or mitral stenosis on baseline TTE; and (4) No prior mitral or tricuspid valve surgeries at the time of both the baseline and last TTEs (Figure S1 in Multimedia Appendix 1).

We divided our cohort into two groups: (1) the telehealth group, consisting of patients who received telehealth services for at least two weeks within 90 days of the baseline TTE (patients who received telehealth after their last TTE were excluded to avoid confounding) and (2) the control group, consisting of patients who did not participate in the telehealth program at any point during the follow-up period.

### Ethical Considerations

This single-center retrospective study was approved by the institutional review board (201804072RINA) and conducted by the Taiwan ELEctroHEALTH (TELEHEALTH) study group. Written informed consent was waived due to the retrospective nature of the study. However, all participants had signed telehealth intervention agreements before enrollment. To protect patient privacy and ensure anonymity, all collected data were thoroughly deidentified and replaced with unique study identifiers.

### Telehealth Services

Since 2010, the Telehealth Center of NTUH has been pioneering the use of remote care specifically for patients with CV disease [23-25]. We invited patients admitted to the CV ward at NTUH to participate in our telehealth program; these patients usually presented with conditions such as arrhythmias, acute myocardial infarction (AMI), coronary artery disease (CAD), congestive HF, or a history of surgical or congenital heart defects. Prior to initiating telehealth services, a comprehensive eligibility assessment was conducted. This included a face-to-face training session for both the patient and their primary caregiver. The session focused on the proper operation of sensors, including manometers, oximeters, glucometers, and electrocardiography devices. Notably, detailed instructions were given on proper home blood pressure (BP) measurement techniques, following established guidelines and using commercially available BP monitors.

Participating patients recorded their biometric data daily, including BP, pulse rate, finger-stick blood glucose, single-lead electrocardiogram, and oxygen saturation. All collected data was securely transmitted to a cloud-based database. This centralized platform allowed case managers and physicians to remotely monitor our patients. Upon identifying any concerning measurements, defined as data exceeding or falling below established thresholds or exhibiting other abnormalities, case managers would initiate immediate action. This involved direct contact with patients to verify their well-being, investigate potential issues, and offer guidance on dose adjustments. The comparison of clinical care received between the telehealth and nontelehealth groups is presented in Table S1 in Multimedia Appendix 1.

Case managers, who had attained at least level 2 out of 4 in our center (Table S2 in Multimedia Appendix 1), contacted patients and caregivers every 2-3 days to monitor their overall condition, and more frequently if unstable conditions were present. During the same period (2010-2020), we enrolled control group patients who were admitted to the CV ward, received only standard care, and did not participate in the telehealth program.

### Clinical Data

Baseline demographics, BP, prescribed medications, echocardiographic parameters, and past histories of percutaneous coronary interventions (PCI) were collected. Baseline BP was defined as BP measured within 1 month of baseline TTE. The Charlson Comorbidity Index (CCI) was calculated, excluding data on HIV infection status to comply with confidentiality regulations mandated by the HIV Infection Control and Patient Rights Protection Act. Educational level, number of rehospitalizations due to cardiovascular causes, and number of emergency room visits were manually reviewed from electronic medical records.

### Endpoints

The primary endpoint was defined as MR/TR regression from ≥moderate to < moderate degree. The follow-up period was from baseline TTE to the last TTE. The secondary endpoint was ACD. The follow-up duration was from baseline TTE to the date of ACD or the last follow-up, which ended on December 31, 2022. The date and cause of death were obtained from both electronic records and research data from the National Health Insurance, a government-run, single-payer plan covering over 99% of the population in Taiwan [26].

### Transthoracic Echocardiography

In patients with multiple exams, we used the earliest qualifying TTE as the baseline for analysis (Figure S1 in Multimedia Appendix 1). Trained sonographers performed the TTEs using commercially available equipment. Chamber quantification, including left ventricular ejection fraction (LVEF), left atrial (LA) dimension, LV end-diastolic dimension (LVEDD), and LV end-systolic dimension (LVESD), was done based on guideline recommendations [27]. The severity of MR and TR was quantified comprehensively using semi-quantitative and quantitative methods [28]. To assess MR/TR regression, we reviewed all available TTEs. In patients receiving surgery or transcatheter intervention on the mitral or tricuspid valve, the presurgical TTEs were used as the last TTE. To ensure that the severity of MR and TR was correctly graded, 20 random cases were selected for re-evaluation. The intraclass correlation coefficient (ICC) was calculated, which was 0.85 for both MR and TR. In cases of conflicting severity interpretations, two experienced imagers (LTY and CCH) discussed to reach a final decision.

### Statistical Analysis

Continuous variables, expressed as mean (SD) or median (IQR) according to data distribution, were compared using Student *t* tests. Categorical data, presented as counts and percentages, were compared using chi-square tests and/or Fisher exact test. The primary endpoint of MR or TR regression was analyzed using the Cox proportional hazard model, where variables with clinical relevance plus univariable *P*≤.05 were chosen for multivariable analyses. PCI was treated as a time-dependent variable in the multivariable model. Adjusted cumulative incidence for MR/TR regression and survival were presented using the Kaplan-Meier curves. A linear mixed model with follow-up duration as a fixed effect, random intercepts at the patient level, and random slopes for follow-up duration were used to assess time-dependent changes in TR peak pressure gradient (TRPG) and evaluate its interaction with telehealth intervention. All statistical analyses were performed using commercially available software (JMP 17 and SAS 9.4, SAS Institute Inc., R version 4.1.2, R Foundation). A 2-sided *P*<.05 was considered statistically significant.

## Results

### Baseline Characteristics Between MR Regressors and Nonregressors

**Figure 1:**
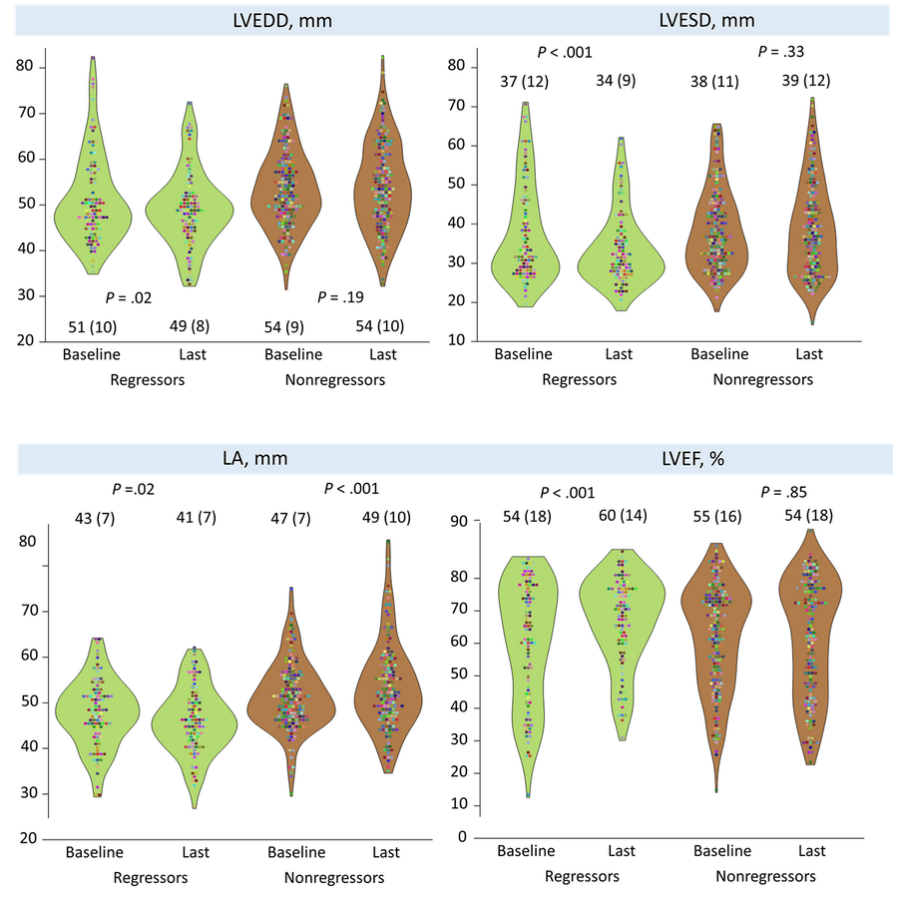
Left heart parameters between regressors and nonregressors. Patients with mitral regurgitation (MR) regression to less than moderate had smaller left ventricular (LV) and left atrial (LA) sizes, as well as improved left ventricular ejection fraction (LVEF) compared with nonregressors. LVEDD: LV end-diastolic dimension; LVESD: LV end-systolic dimension.

The final MR cohort consisted of 264 patients with moderate or greater MR (Table 1). At a median follow-up of 5 (IQR 2.3-7.3) years, there were 97 regressors and 167 nonregressors. As compared with nonregressors, regressors were younger, more likely to participate in the telehealth program, had a higher level of education, smaller LA dimension, and less severe baseline MR (all *P*≤.004); both groups exhibited similar mechanisms of MR, TR severity, peak TRPG, and medication usage (*P*≥.06). At last TTE, as expected, regressors had smaller LA/LV dimensions, less severe TR, and better LVEF (all *P*≤.005; Table 1 and Figure 1). At a median of 6.8 (IQR 2.3-10.2) years, 62 patients underwent PCI; regressors had 1.69-fold likelihood of having PCI (age- and sex-adjusted hazard ratio [HR], 1.69; 95% CI [29], 1.02-2.80, *P*=.04) as compared with nonregressors.

**Table 1:** Baseline characteristics of mitral regurgitation regressors versus nonregressors and telehealth versus nontelehealth groups (N=264).

Characteristics			Regressors^a^ (n=97)		Nonregressors^b^ (n=167)		*P* value		Telehealth (n=74)			Nontelehealth (n=190)		*P* value
Age (years), mean (SD)			64 (14)		69 (13)		.002		64 (14)			68 (13)		.03
Male, n (%)			41 (42)		82 (49)		.28		41 (55)			100 (53)		.68
Educational level≥ high school^c^, n (%)			57 (68)		67 (50)		.006		47 (72)			77 (50)		.002
SBP^d^ (mm Hg), mean (SD)			130 (23)		133 (25)		.22		128 (23)			133 (25)		.13
DBP^e^, mm Hg, mean (SD)			76 (14)		76 (16)		.97		76 (14)			189 (16)		.85
Telehealth, n (%)			39 (40)		35 (20)		<.001		—^f^			—		—
AFib^g^ at TTE^h^, n (%)			18 (19)		46 (28)		.09		16 (6)			58 (21)		.53
CCI^i^, mean (SD)			1.26 (1.44)		1.20 (1.44)		.72		1.22 (1.17)			1.22 (1.53)		.98
Hypertension, n (%)			30 (31)		56 (34)		.66		26 (35)			60 (32)		.58
Diabetes mellitus, n (%)			25 (25)		49 (29)		.53		19 (26)			55 (29)		.59
MI^j^, n (%)			9 (9)		17 (10)		.81		9 (12)			17 (9)		.43
Heart failure, n (%)			31 (31)		50 (29)		.73		29 (39)			52 (27)		.06
Malignancy, n (%)			6 (6)		9 (5)		.78		5 (7)			10 (5)		.64
Statin, n (%)			32 (33)		51 (31)		.67		29 (39)			54 (28)		.09
Antiplatelet, n (%)			63 (65)		94 (56)		.16		49 (66)			108 (57)		.16
Anticoagulant, n (%)			35 (36)		42 (25)		.06		31 (42)			46 (24)		.005
ACEi^k^ and ARB^l^, n (%)			65 (67)		111 (66)		.92		47 (64)			112 (59)		.49
Beta-blocker, n (%)			68 (70)		105 (63)		.23		59 (80)			115 (60)		.001
CCB^m^, n (%)			37 (38)		62 (37)		.86		24 (32)			75 (40)		.28
Diuretics, n (%)			59 (61)		117 (70)		.13		52 (70)			124 (65)		.43
**Baseline echocardiographic parameters**														
	LVEF^n^ (%), mean (SD)	53 (18)		55 (16)		.50				50 (1)	56 (16)		.01	
	LA^o^ dimension (cm), mean (SD)	4.3 (0.7)		4.6 (0.7)		<.001				4.3 (0.7)	4.6 (0.7)		.002	
	LVEDD^p^ (mm), mean (SD)	51 (10)		54 (9)		.08				53 (9)	53 (9)		.86	
	LVESD^q^ (mm), mean (SD)	37 (12)		38 (11)		.62		40 (13)			37 (11)		.14	
**Mechanisms of MR^r^**							.09							.05
	FMR^s^, n (%)	85 (89)		134 (81)				66 (90)			153 (81)			
	Primary MR, n (%)	11 (11)		32 (19)				7 (10)			36 (19)			
**Baseline MR**							.004							<.001
	Moderate, n (%)	84 (86)		116 (69)				67 (91)			133 (70)			
	Moderate-severe, n (%)	12 (12)		45 (26)				7 (9)			50 (26)			
	Severe, n (%)	1 (1)		6 (3)				0 (0)			7 (4)			
**Baseline TR^t^**							.09							.89
	None, n (%)	1 (1)		0 (0)				0 (0)			1 (<1)			
	Trivial, n (%)	1 (1)		1 (<1)				1 (<1)			1 (1)			
	Mild, n (%)	39 (40)		50 (30)				28 (38)			61 (32)			
	Mild-moderate, n (%)	14 (14)		26 (16)				12 (16)			28 (15)			
	Moderate, n (%)	37 (38)		68 (41)				27 (36)			78 (41)			
	Moderate-severe, n (%)	5 (5)		17 (10)				5 (7)			17 (9)			
	Severe, n (%)	0 (0)		5 (3)				1 (<1)			4 (2)			
	Baseline TR ≥moderate, n (%)	42 (43)		90 (54)		.09		33 (45)			99 (52)		.27	
	Baseline TRPG^u^ (mm Hg), mean (SD)	35 (13)		36 (12)		.40		35 (12)			36 (12)		.64	

^a^MR regressors: patients with MR regression to less than moderate severity in the last transthoracic echocardiography.

^b^Nonregressors: patients with MR severity equal to or more than moderate in the last transthoracic echocardiography.

^c^In 47 patients, the educational levels were unknown.

^d^SBP: systolic blood pressure.

^e^DBP: diastolic blood pressure.

^f^Not available.

^g^TRPG: tricuspid regurgitation peak gradient.

^h^AFib: atrial fibrillation.

^i^CCI: Charlson comorbidity index.

^j^MI: myocardial infarction.

^k^ACEi: angiotensin-converting enzyme inhibitor.

^l^ARB: angiotensin receptor blocker.

^m^CCB: calcium channel blocker.

^n^LVEF: left ventricular ejection fraction.

^o^LA: left atrial.

^p^LVEDD: LV end-diastolic dimension.

^q^LVESD: LV end-systolic dimension.

^r^MR: mitral regurgitation (in 2 patients, the MR mechanisms were unknown).

^s^FMR: functional mitral regurgitation.

^t^TR: tricuspid regurgitation.

^u^TRPG: tricuspid regurgitation peak gradient.

### Telehealth Versus Nontelehealth Patients in the Mitral Regurgitation Cohort

Compared to the nontelehealth group, telehealth patients were younger, had a higher level of education, smaller LA dimensions, fewer cases of ≥moderate-severe MR, and lower baseline LVEF; this was reflected in their higher likelihood of being treated with anticoagulants and beta-blockers (all *P*≤.04; Table 1). At the final TTE, telehealth participants had smaller LA dimensions (*P*=.003), less ≥moderate TR (*P*=.008), and lower TRPG (*P<*.001) yet similar LVEF and LV dimensions (all *P*≥.19; Table 2). Telehealth participants also experienced fewer emergency room visits and rehospitalizations for cardiovascular causes during follow-up (Table 2). The time elapsed from baseline TTE to last TTE in the telehealth and nontelehealth group (mean 4.6, SD 2.8 years vs mean 5.3, SD 3.1 years; *P*=.09) was similar. Between baseline and the last TTE, the telehealth and nontelehealth group had similar numbers of follow-up TTEs (mean 5.7, SD 4.6 vs mean 6.0, SD 3.8 times; *P*=.62). The linear mixed model revealed a significant time-dependent increase in TRPG (+0.03 mmHg per month, *P*=.01) in the nontelehealth group, while a significant interaction between telemedicine and follow-up duration (–0.09 mm Hg per month, *P*<.001) suggests that the telehealth group experienced a modest but significant monthly TRPG decrease (–0.05 mm Hg per month; Figure S2 in Multimedia Appendix 1). At a median follow-up of 6.8 (IQR 2.3-10.2) years, the telehealth group had 1.79-fold likelihood of having PCI (age- and sex-adjusted hazard ratio 1.79, 95% CI 1.28-2.50, *P*<.001) as compared with nontelehealth participants.

**Table 2:** Follow-up characteristics of mitral regurgitation regressors versus nonregressors and telehealth versus nontelehealth groups (N=264).

Characteristics				Regressors (n=97)		Nonregressors (n=167)		*P* value		Telehealth (n=74)	Nontelehealth (n=190)			*P* value		
**Echocardiographic parameters at last TTE**																
	LVEF^a^ (%), mean (SD)			60 (13)		55 (18)		.005		54 (17)			57 (16)			.19
	LA^b^ dimension (mm), mean (SD)			4.1 (0.7)		4.9 (0.9)		<.001		4.3 (0.8)			4.7 (1.0)			.003
	LVEDD^c^ (mm), mean (SD)			49 (8)		54 (10)		<.001		53 (10)			52 (9)			.52
	LVESD^d^ (mm), mean (SD)			34 (9)		39 (12)		<.001		38 (12)			36 (11)			.24
	TR^e^≥ moderate, n (%)			19 (20)		108 (65)		<.001		26 (35)			101 (53)			.008
	TRPG^f^ (mm Hg), mean (SD)			35 (13)		36 (12)		.40		31 (13)			38 (17)			<.001
	PCI^g^ after baseline, n (%)			29 (30)		33 (20)		.06		20 (27)			42 (22)			.40
**Follow-up events**																
		ER^h^ visit, number, mean (SD)	2.0 (5.1)		1.9 (4.5)		.83		1.4 (2.0)			2.2 (5.4)			.10	
		CV^i^-related admission, mean (SD)	1.8 (1.9)		1.9 (2.3)		.79		1.3 (1.7)			2.1 (2.2)			.003	

^a^LVEF: left ventricular ejection fraction.

^b^LA: left atrial.

^c^LVEDD: LV end-diastolic dimension.

^d^LVESD: LV end-systolic dimension.

^e^TR: tricuspid regurgitation.

^f^TRPG: tricuspid regurgitation peak gradient.

^g^PCI: percutaneous coronary interventions.

^h^ER: emergency room.

^i^CV: cardiovascular.

### Primary Endpoint: MR Regression to <Moderate Degree

**Figure 2:**
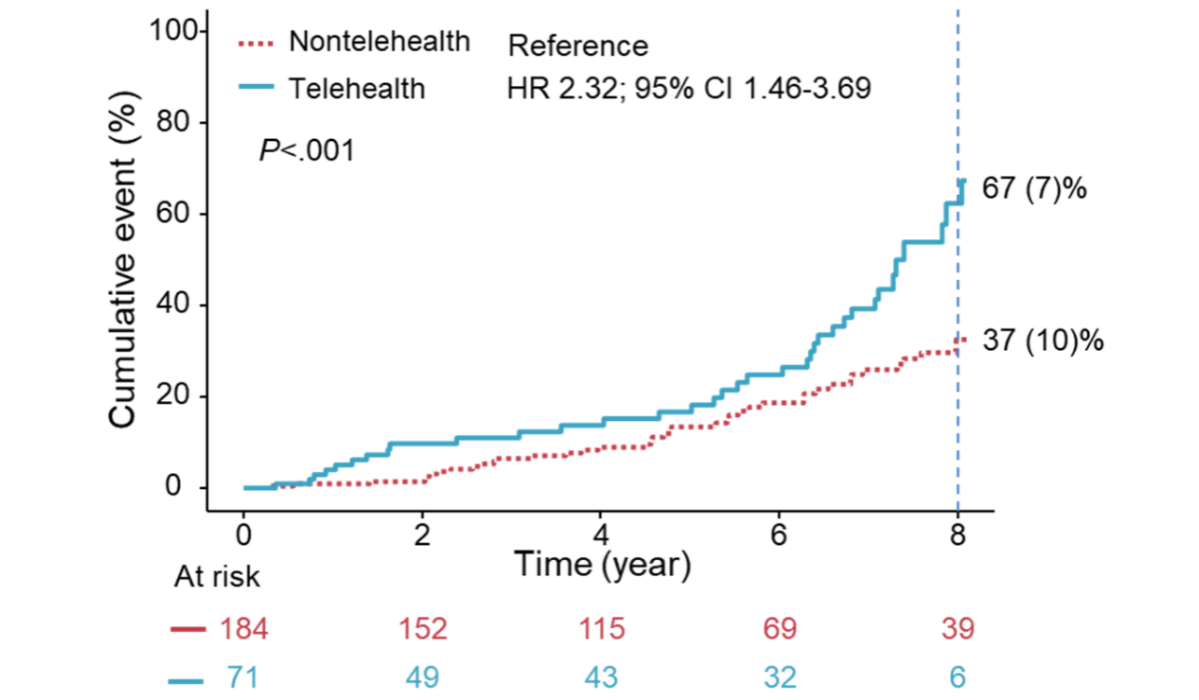
The cumulative incidence for mitral regurgitation (MR) regression. Kaplan-Meier curves, adjusted for age, sex, left ventricular ejection fraction (LVEF), left atrial (LA) size, and baseline MR/TR severity, revealed that the telehealth group had a higher 8-year incidence of MR regression to less than moderate. MR: mitral regurgitation; HR: hazard ratio.

In univariable analysis, smaller baseline LA dimensions, lower LVEF, less severe baseline MR/TR, performance of PCI, prescription of beta-blockers, and the telehealth service were associated with MR regression (all *P*≤.05; Table 3). A comparison of patients with or without beta-blocker use was shown in Table S3 in Multimedia Appendix 1. Those who used beta-blockers were older, had higher systolic BP, more prevalent hypertension, and greater use of concomitant renin-angiotensin system inhibitors and antiplatelet agents; they also had lower LVEF and larger LA size compared to nonusers (all *P*≤.04). Multivariable analysis adjusted for MR mechanisms and abovementioned parameters, including time-dependent PCI, revealed that telehealth group was the only determinant for MR regression (*P*=.001; Table 3). An additional multivariable model excluding the “telehealth group” revealed that the use of beta-blockers was marginally associated with MR regression (Table S4 in Multimedia Appendix 1). Adjusted Kaplan-Meier curves revealed that the telehealth group had a higher 8-year incidence of MR regression to <moderate (mean 67, SD 7% vs mean 37, SD 10%; *P*<.001; Figure 2). The incidence of MR regression to <moderate was 11.4 (95% CI, 8.1-15.6) per 100-person years in the telehealth group and 5.8 (95% CI, 4.4-7.5) per 100-person years in the nontelehealth group. In a subgroup analysis including only those with baseline moderate MR, the telehealth group remained independently associated with MR regression to <moderate (HR 2.56, 95% CI 1.56–4.21; *P*<.001; N=200; Table S5 in Multimedia Appendix 1). Also, when we set the last follow-up TTE before the pandemic outbreak in Taiwan (May 2021), multivariable analysis consistently shows the link between telehealth intervention and MR regression (Table S6 in Multimedia Appendix 1). For the telehealth-subgroup analysis, the duration of telehealth participation was not associated with MR regression (hazard ratio, 0.99; 95% CI, 0.99-1.00; *P*=.10).

**Table 3:** Univariable and multivariable determinants for mitral regurgitation regression to less than moderate (N=97).

Variables		Univariable analysis		Multivariable analysis	
		Hazard ratio (95% CI)	*P* value	Hazard ratio (95% CI)	*P* value
Telehealth vs nontelehealth		2.67 (1.72-4.12)	<.001	2.20 (1.35-3.58)	.001
Age (years)		0.99 (0.98-1.00)	.46	0.99 (0.98-1.01)	.94
Male		1.14 (0.76-1.71)	.51	1.08 (0.69-1.68)	.72
SBP^a^, mm Hg		1.00 (0.99-1.01)	.88	—^b^	—
DBP^c^, mm Hg		0.99 (0.98-1.01)	.97	—	—
CCI^d^		1.07 (0.93-1.21)	.30	—	—
AFib^e^ at TTE^f^		0.71 (0.42-1.19)	.18	—	—
ACEi^g^ and ARB^h^		0.93 (0.60-1.42)	.74	—	—
Diuretics		1.18 (0.78-1.78)	.41	—	—
Statin		1.19 (0.77-1.82)	.42	—	—
Antiplatelets		1.14 (0.75-1.74)	.51	—	—
Beta-blocker		1.53 (0.99-2.37)	.049	1.32 (0.81-2.15)	.26
CCB^i^		1.11 (0.73-1.68)	.61	—	—
Baseline LA^j^ dimension, cm		0.73 (0.55-0.97)	.03	0.78 (0.57-1.07)	.13
Baseline LVEF^k^, %		0.98 (0.97-0.99)	.04	0.99 (0.97-1.00)	.25
Baseline LVEDD^l^, mm		0.98 (0.96-1.01)	.36	—	—
Baseline LVESD^m^, mm		1.00 (0.98-1.02)	.47	—	—
TRPG^n^, mm Hg		1.00 (0.98-1.02)	.53	—	—
**Baseline MR^o^ severity (Ref: moderate)**					
	Moderate-severe	0.51 (0.27-0.93)	.02	0.63 (0.31-1.25)	.18
	Severe	0.24 (0.03-1.84)	.17	0.40 (0.05-3.25)	.39
	Primary MR vs FMR^p^	0.78 (0.41-1.47)	.42	1.34 (0.67-2.68)	.40
	Baseline TR^q^<moderate	1.50 (0.99-2.27)	.05	1.28 (0.83-2.00)	.25
	Time-dependent PCI^r^	1.75 (1.13-2.71)	.01	1.23 (0.76-1.99)	.38

^a^SBP: systolic blood pressure.

^b^Not applicable.

^c^DBP: diastolic blood pressure.

^d^CCI: Charlson Comorbidity Index.

^e^AFib: atrial fibrillation.

^f^TTE: transthoracic echocardiography.

^g^ACEi: angiotensin-converting enzyme inhibitor.

^h^ARB: angiotensin receptor blocker.

^i^CCB: calcium channel blocker.

^j^LA: left atrial.

^k^LVEF: left ventricular ejection fraction.

^l^LVEDD: LV end-diastolic dimension.

^m^LVESD: LV end-systolic dimension.

^n^TRPG: tricuspid regurgitation peak gradient.

^o^MR: mitral regurgitation

^p^FMR: functional mitral regurgitation.

^q^TR: tricuspid regurgitation.

^r^PCI: percutaneous coronary interventions.

### Secondary Endpoint: Determinants for ACD

**Figure 3:**
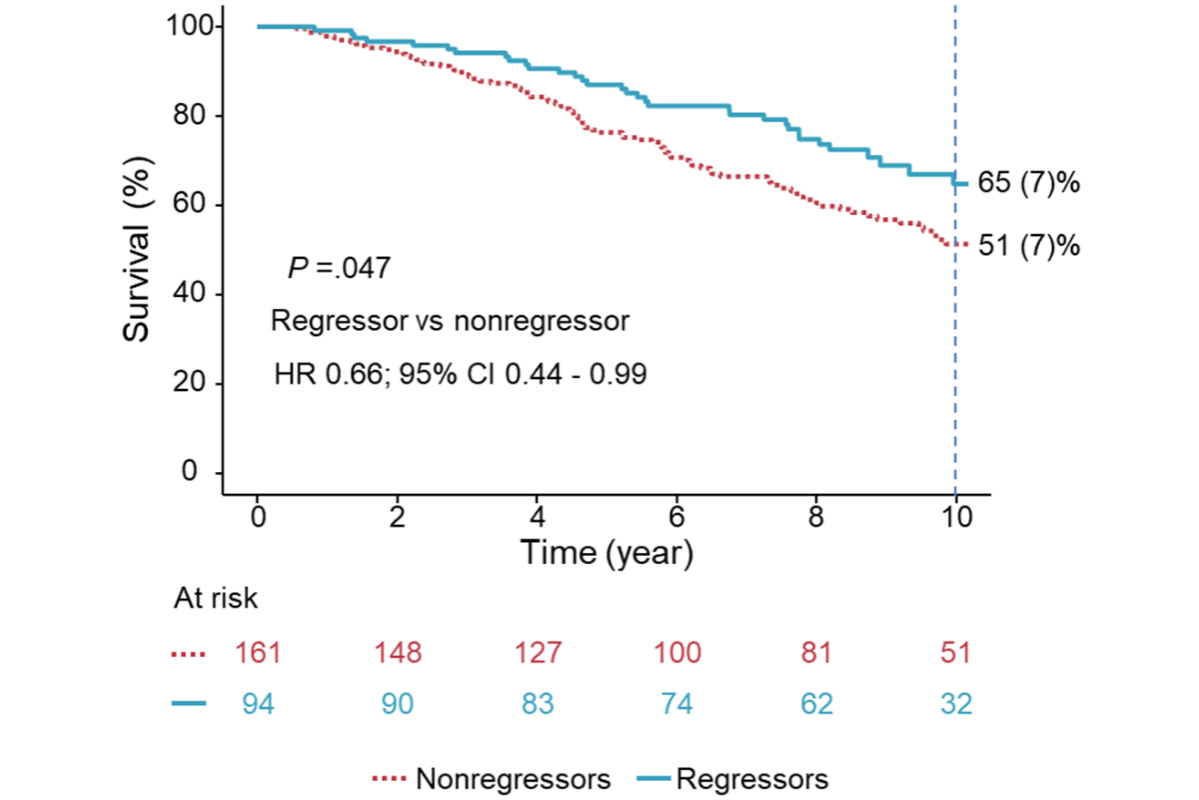
The survival curves between regressors and nonregressors. Kaplan-Meier curves, adjusted for the same covariates, revealed that regressors had better 10-year survival compared with nonregressors. HR: hazard ratio.

**Figure 4:**
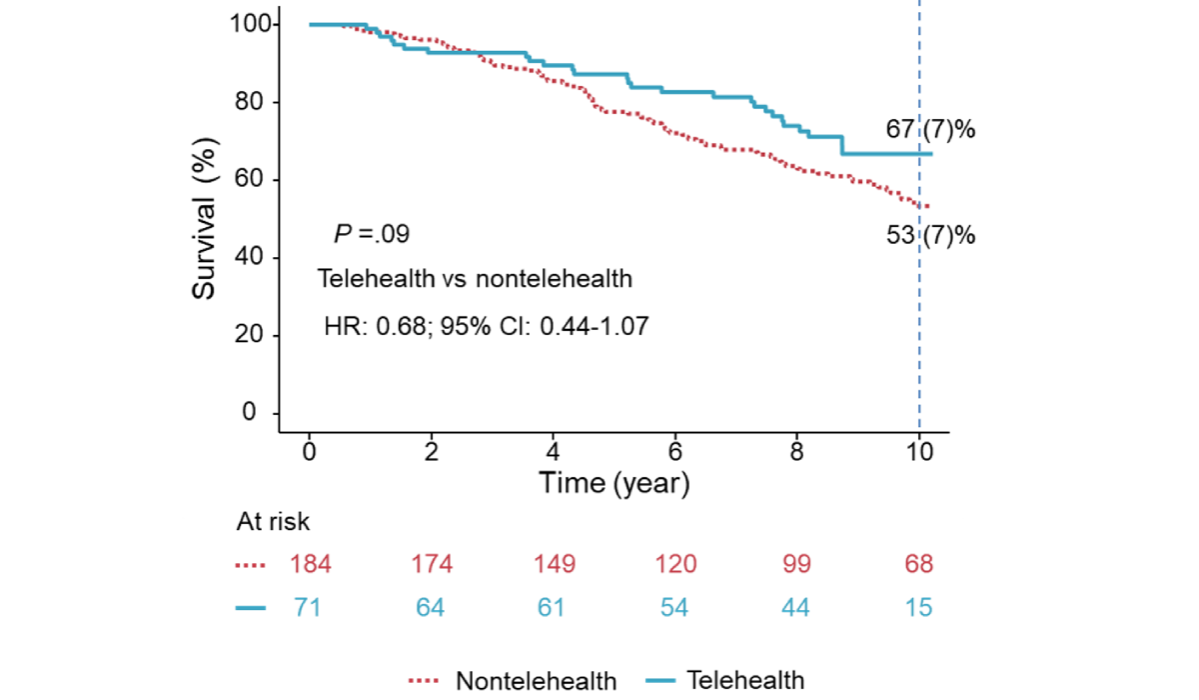
The survival curves between the telehealth and nontelehealth groups. The telehealth group tended to have better 10-year survival than the nontelehealth group, as shown by adjusted Kaplan-Meier curves. HR: hazard ratio.

As of December 31, 2022, the follow-up rate for ACD was 100%. Over a median follow-up of 8.5 (IQR 4.8-10.7) years, 134 (51%) deaths occurred in 264 patients, with a 10-year survival rate of 51 (3%). Univariable determinants for ACD were older age, lower diastolic BP, use of diuretics and calcium channel blocker, higher CCI, larger baseline LA size, reduced LVEF, performance of PCI, and regression of MR to <moderate (all *P*≤.03; Table 4). Age-adjusted multivariable determinants for ACD-free survival were MR regressors (HR 0.61, 95% CI 0.41-0.92, *P*=.02), better LVEF (HR per 1%, 0.97, 95% CI 0.96-0.98, *P*<.001), and performance of PCI (HR 0.82, 95% CI 0.77-0.88, *P*<.001) (Table 4). Adjusted Kaplan-Meier curves showed that regressors had better 10-year survival as compared with nonregressors (*P*=.047) (Figure 3). After adjusting for the same covariates, the telehealth group tended to have better 10-year survival than the nontelehealth group (*P*=.09; Figure 4).

**Table 4:** Univariable and multivariable determinants for all-cause death (N=134) in the mitral regurgitation cohort.

Variables		Univariable analysis				Multivariable analysis			
		Hazard ratio (95% CI)		*P* value		Hazard ratio (95% CI)		*P* value	
Regressors vs nonregressors		0.60 (0.41-0.88)		.007		0.61 (0.41-0.92)		.02	
Age (years)		1.05 (1.03-1.06)		<.001		1.05 (1.03-1.07)		<.001	
Male		0.96 (0.68-1.35)		.82		0.86 (0.60-1.24)		.43	
SBP^a^, mm Hg		1.00 (0.99-1.01)		.19		—^b^		—	
DBP^c^, mm Hg		0.98 (0.97-0.99)		.01		0.99 (0.98-1.01)		.80	
Telehealth vs nontelehealth		0.73 (0.48-1.11)		.14		—		—	
CCI^d^		1.18 (1.06-1.30)		.002		1.11 (0.99-1.25)		.06	
AFib^e^ at TTE^f^		1.04 (0.70-1.54)		.84		—		—	
ACEi^g^ and ARB^h^		1.21 (0.83-1.76)		.29		—		—	
Diuretics		1.78 (1.20-2.63)		.002		0.99 (0.64-1.52)		.96	
Statin		0.98 (0.68-1.42)		.94		—		—	
Antiplatelets		1.26 (0.88-1.79)		.18		—		—	
Beta-blocker		1.18 (0.82-1.70)		.35		—		—	
CCB^i^		1.44 (1.03-2.03)		.03		1.03 (0.71-1.50)		.85	
Baseline LA^j^ dimension, cm		1.26 (1.02-1.57)		.03		1.18 (0.91-1.54)		.20	
Baseline LVEF^k^, %		0.98 (0.97-0.99)		.01		0.97 (0.96-0.98)		<.001	
Baseline LVEDD^l^, mm		1.01 (0.99-1.03)		.22		—		—	
Baseline LVESD^m^, mm		1.01 (0.99-1.02)		.06		—		—	
TRPG^n^, mm Hg		1.01 (0.99-1.02)		.05		—		—	
**Baseline MR severity (Ref: moderate)**									
	Moderate-severe		0.84 (0.55-1.28)		.42		0.89 (0.55-1.45)		.66
	Severe		0.88 (0.32-2.41)		.81		1.15 (0.35-3.73)		.81
	Primary MR^o^ vs FMR^p^		0.72 (0.44-1.17)		.17		—		—
	Baseline TR^q^<moderate		0.98 (0.70-1.38)		.93		1.24 (0.86-1.80)		.23
	Time-dependent PCI^r^		0.81 (0.77-0.86)		<.001		0.82 (0.77-0.88)		<.001

^a^SBP: systolic blood pressure.

^b^Not applicable.

^c^DBP: diastolic blood pressure.

^d^CCI: Charlson Comorbidity Index.

^e^AFib: atrial fibrillation.

^f^TTE: transthoracic echocardiography.

^g^ACEi: angiotensin-converting enzyme inhibitor.

^h^ARB: angiotensin receptor blocker.

^i^CCB: calcium channel blocker.

^j^LA: left atrial.

^k^LVEF: left ventricular ejection fraction.

^l^LVEDD: LV end-diastolic dimension.

^m^LVESD: LV end-systolic dimension.

^n^TRPG: tricuspid regurgitation peak gradient.

^o^MR: mitral regurgitation.

^p^FMR: functional mitral regurgitation.

^q^TR: tricuspid regurgitation.

^r^PCI: percutaneous coronary interventions.

### Baseline Characteristics Between TR Regressors and Nonregressors

In the TR cohort, which included 245 patients with ≥moderate TR at baseline, there were 87 regressors and 158 nonregressors, with a median follow-up of 4.99 (IQR 2.57-7.25) years (Table S7 in Multimedia Appendix 1). Compared to nonregressors, regressors were younger, had smaller baseline LA dimensions, less severe TR, and were more likely to receive telehealth services (all *P*≤.05). At last, TTE regressors had higher LVEF, smaller LA dimension, LVESD, and less severe MR (all *P*≤.05).

### Baseline Characteristics Between Telehealth and Nontelehealth Patients in the TR Cohort

Compared to the nontelehealth group (Table S7 in Multimedia Appendix 1), telehealth patients were younger, had lower LVEF and smaller LA dimensions (all *P*≤.02), with similar baseline MR/TR severity. At the final TTE, the telehealth group had a smaller LA dimension (*P*<.001).

### Determinants of TR Regression to <Moderate Degree

Univariable predictors of TR regression were the prescription of beta-blockers, smaller LA dimension, less severe baseline TR, performance of PCI, and the telehealth group (all *P*≤.05; Table S8 in Multimedia Appendix 1). In multivariable analysis, beta-blocker use and more severe baseline TR were robust markers of TR regression (all *P*≤.048); telehealth participation was not a multivariable determinant (*P*=.33; Table S8 in Multimedia Appendix 1).

### Determinants of ACD in TR Cohort

At a median follow-up of 8.6 (IQR 5.2-11.0) years, 113 (46%) ACD occurred in 245 patients, with a 10-year survival rate of 54 (3%). Univariable determinants for ACD in the TR cohort were shown in Table S9 in Multimedia Appendix 1; the telehealth group was associated with better survival (HR 0.5; *P*=.005). However, in multivariable analysis, only younger age (*P*<.001) and better LVEF (*P*=.004) were associated with ACD (Table S9 in Multimedia Appendix 1).

## Discussion

### Overview

To the best of our knowledge, this is the first study to investigate the impact of telemedicine on the regression of MR or TR. Our principal findings were (1) MR and TR regressors were younger, more likely to participate in the telehealth program, had higher educational levels, used more beta-blockers, had smaller LA, reflected by less severe baseline MR or TR, and as expected, had better chamber reverse remodeling at last TTE. Interestingly, MR regressors had less severe TR at last TTE; likewise, TR regressors also had less severe MR at last TTE; (2) enrollment in the telehealth program was a robust indicator for MR regression in the entire cohort and in patients with baseline moderate MR, even after accounting for the effect of COVID outbreak; however, its effect on TR regression was less pronounced; (3) the incidence of MR regression to <moderate (MR regressors) was 11.4 (95% CI 8.1-15.6) per 100-person years in the telehealth group; (4) the telehealth group had fewer emergency room visits and rehospitalizations for cardiovascular causes; (5) besides younger age, better LVEF, and the performance of PCI, MR regressors independently linked to better survival; (6) TR regression was associated with the prescription of beta-blockers and with less severe TR at baseline; and (7) in the TR cohort, independent determinants of ACD included older age and reduced LVEF; TR regression was not linked to ACD.

### Benefits of Telehealth and the Unmet Need

Telehealth has emerged as a promising healthcare model with the potential to improve outcomes over a variety of disciplines, including chronic CV diseases. It has been shown to reduce HF hospitalization and mortality [14-16], and when operated by a nurse practitioner, it was noninferior to cardiologist-led standard care in patients with AMI [30]. Indeed, our study found that, although patients in both the MR and TR cohorts showed overall reduced survival (10-year survival rate of 51-54%)—a trend previously noted in patients with functional MR [31], functional TR [32], and heart failure with preserved LVEF [33]—telehealth intervention emerged as the sole determinant of MR regression; notably, MR regression served as a strong marker for improved survival (Tables 3 and 4). Additionally, telehealth has demonstrated better cost-effectiveness when considering the reduction in subsequent hospitalizations [25].

However, data on the impact of telemedicine on VHD remain scarce, with most studies focusing on patients undergoing transcatheter aortic valve replacement for aortic stenosis [22,34], and only one study reporting associations with MR and TR progression [23].

### Factors Associated With Regression of MR or TR

In our MR-cohort (Tables 1 and 2), we found that univariable determinants of MR regression included telehealth participation, beta-blocker use, lower LVEF, smaller LA dimension, less severe baseline MR/TR, as well as the performance of PCI; MR regressors also had improved LVEF and further reductions in LV and LA sizes. These findings were supported by several studies. Campwala et al [35] found that in patients undergoing coronary artery bypass grafting, postsurgical MR regression was associated with reductions in LV dimensions, improved LVEF, and the use of beta-blockers. Likewise, Bartko et al [36] found that larger LA size and concomitant TR were associated with MR progression. These observations are unsurprising, as coronary revascularization is associated with reverse cardiac remodeling, which improves MR through enhanced coaptation of the mitral leaflets [10]. On the other hand, the use of beta-blockers, incorporated as part of the guideline-directed medical therapy in HF with reduced LVEF [37], was associated with MR regression, possibly due to myocardial protection and promotion of reverse cardiac remodeling [35]; the effect of beta-blockers on MR regression remained evident, albeit with marginal statistical significance, after excluding “telehealth” in the multivariable analysis (Table S4 in Multimedia Appendix 1). In the final multivariable analysis, however, only “telehealth” was linked to MR regression (Table 3). Potential mechanisms for this association will be discussed later.

The COVID-19 pandemic represented a major external factor influencing contemporary clinical studies. Despite the challenges, our hospital’s telemedicine services continued without significant disruption, unlike standard outpatient clinics. To minimize confounding effects, we excluded the follow-up period corresponding to the COVID-19 lockdown in Taiwan (since May 2021), and telehealth remained a significant independent determinant of MR regression (Table S6 in Multimedia Appendix 1), further underscoring its efficacy and resilience in providing consistent care during crises.

Univariable determinants of TR regression in this study included telehealth participation, beta-blocker use, smaller LA dimensions, less severe baseline TR, and performance of PCI. Given the similarities between these factors and those observed in MR regression, we hypothesized that LV and LA reverse remodeling likely plays a pivotal role in TR regression as well. The persistent significance of beta-blocker use in the multivariable analysis suggests that left heart function may play an even more crucial role in influencing TR regression than previously anticipated.

### The Role of Telehealth in MR/TR Regression

**Figure 5:**
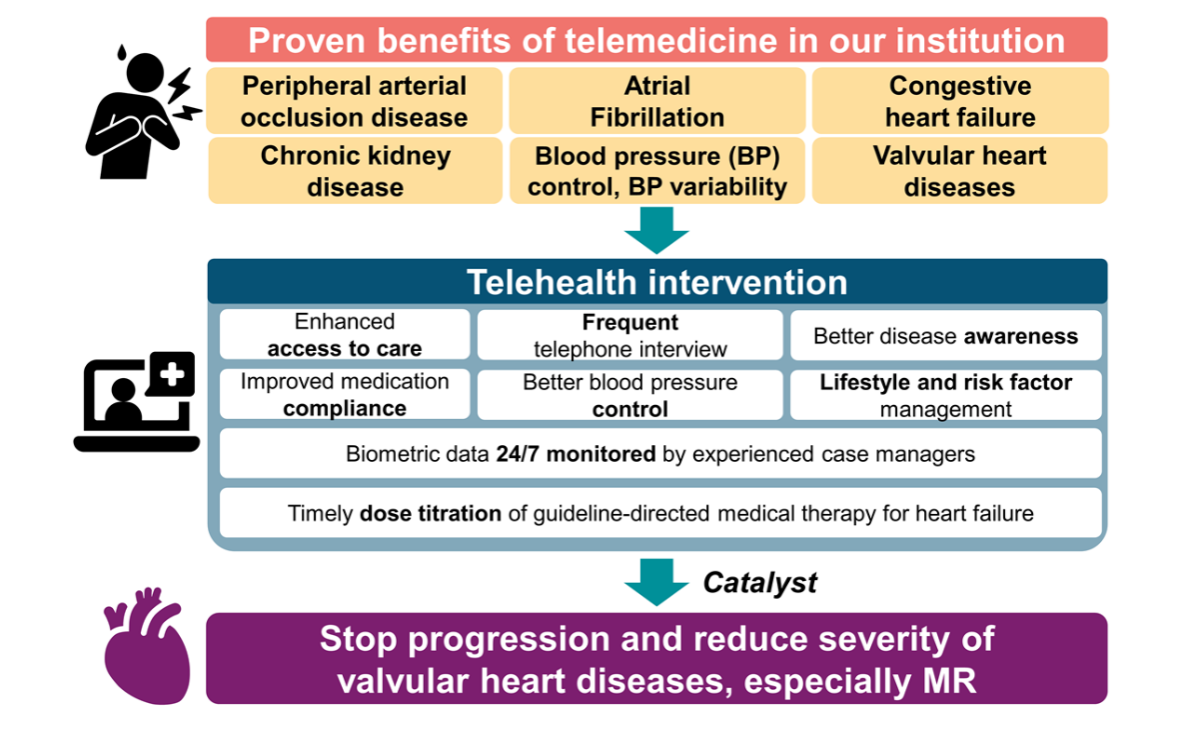
Proven benefits of telemedicine and the potential effects of telehealth on mitral regurgitation (MR) regression. We previously demonstrated the associations between telemedicine and improved outcomes in various diseases through publications from our institution, as well as the services that telemedicine provides. These potential effects may contribute to reverse cardiac remodeling and MR regression [24,25,39-41,46-49].

Previous telehealth studies, including randomized controlled trials [15,16], meta-analysis [38], and studies from our center [24,25,39-41], have demonstrated the benefits of telehealth in reducing mortality, overall medical costs, and rehospitalization, as well as improving blood pressure control. Possible mechanisms include enhanced access to care, optimized risk-factor management, improved medication adherence [42-45], timely dose titration of guideline-directed medical therapy for heart failure, early detection of abnormal events through biometric monitoring, and increased patient awareness through frequent communication with experienced nurse practitioners (Table S1 and S2 in Multimedia Appendix 1 and Figure 5 [24,25,39-41,46-49]) [14,44]. In our center, telephone interviews routinely included questions about medication adherence (“Are you taking your medication regularly?” “Have you experienced any side effects or issues with the medication?”), troubleshooting technical issues with telehealth services (“Are there any problems with the app or monitoring devices?”), and discussing potential dose adjustments. These adjustments were guided by telehealth center physicians and informed by ongoing trends in biodata collected through the program. The current study reveals an independent association between telehealth and MR regression. While causality may be multifaceted and influenced by unmeasured confounding factors, we believe that the aforementioned mechanisms of telehealth intervention may act as a “booster” or “catalyst” in facilitating MR regression. Additionally, the greater use of beta-blockers in the telehealth group may contribute to chamber reverse remodeling, leading to MR regression [37]. The observed lower rehospitalization rates and decrease in TRPG over time may, to some extent, be attributed to MR regression (Table 1 and Figure S2 in Multimedia Appendix 1).

However, given the retrospective nature of this study, unmeasured factors associated with telehealth participation may also influence both telehealth engagement and MR regression. For instance, socioeconomic status has been shown to influence patients’ willingness to participate in telehealth [50]. However, in our telehealth center, all enrolled patients received a 2-week complimentary trial of the telehealth service (Table S1 in Multimedia Appendix 1), ensuring that economic status did not influence participation. Furthermore, the lack of association between the duration of telehealth participation and MR regression suggests that longer participation—and thus the financial commitment—was not necessarily linked to MR regression. However, the higher education level in the telehealth group (Table 1), consistent with previous studies [51], suggests that digital literacy may influence the decision to participate in telehealth services. Whether digital literacy indirectly contributes to MR regression remains to be determined in future studies.

In other words, telehealth programs provide both direct and indirect benefits that enhance existing medical care, potentially facilitating reverse cardiac remodeling [52] and MR regression. Our previous study [23] suggested that telehealth could potentially slow the progression of MR and TR, further supporting its role in mitigating cardiac remodeling. While the precise mechanisms remain incompletely understood, the observed association between telehealth and MR/TR regression highlights its potential as a valuable intervention in patients with VHD (Figure 5 [24,25,39-41,46-49]).

### Clinical Implications

Our investigation distinguishes itself from these contemporary studies into telehealth interventions by being, to our knowledge, the first to examine the impact of telemedicine on MR or TR regression. The latest guideline for the management of lower extremity peripheral artery disease (PAD) [53] recommends the use of telemedicine in patient care, drawing from a recent study conducted by us, which found that PAD patients in our telehealth program exhibited a lower risk of ischemic stroke compared to usual care [24]. Guidelines for the management of valvular and structural heart diseases developed during the COVID-19 pandemic also recommended the use of telemedicine to monitor patients with severe MR [29]. Therefore, it appears that telehealth services are gaining traction as a supplementary treatment for CV diseases, owing to their beneficial effects on patient outcomes. Our study results not only open the door for further research but also support the incorporation of telemedicine into future guidelines for managing patients with VHD, particularly those with significant MR or TR. However, a substantial increase in enrolled patients could lead to manpower shortages in telecare. In such cases, integrating artificial intelligence could enhance clinical decision-making efficiency, provided that legal and ethical concerns are addressed [54].

### Limitations

This research has several limitations. As a retrospective study from a tertiary referral center, it inherently carries the risk of selection bias and unmeasured confounders. In addition, quantitative measurements for assessing MR and TR severity were incomplete. The duration of enrollment in the telehealth program varied among participants, although it was not associated with MR regression herein. Limited access to data on dosage adjustments and patient medication adherence also posed constraints. Regarding cardiac reverse remodeling parameters, data on LV volume and LV global longitudinal strain were lacking. Furthermore, we acknowledge the potential for selection bias among telehealth patients due to disparities in digital literacy and access, which are complex and multifaceted.

### Conclusions

This study is the first to report associations between telehealth services and MR or TR regression. Patients in the telehealth group were 2.2 times more likely to experience MR regression. In addition, MR regressors demonstrated better survival and reverse cardiac remodeling than nonregressors. These findings support integrating telemedicine into the management of moderate or greater MR, which may have important implications for future guidelines.

## Appendix

Multimedia Appendix 1 Flowchart, nurse-level classification, and additional tables of mitral regurgitation and tricuspid regurgitation cohorts.

## Abbreviations

ACD: all-cause death
AMI: acute myocardial infarction
BP: blood pressure
CAD: coronary artery disease
CCI: Charlson Comorbidity Index
CV: cardiovascular
HF: heart failure
HR: hazard ratio
ICC: intraclass correlation coefficient
LA: left atrial
LV: left ventricular
LVEDD: left ventricular end-diastolic dimension
LVEF: left ventricular ejection fraction
LVESD: left ventricular end-systolic dimension
MR: mitral regurgitation
NTUH: National Taiwan University Hospital
PAD: peripheral artery disease
PCI: percutaneous coronary interventions
TR: tricuspid regurgitation
TRPG: tricuspid regurgitation pressure gradient
TTE: transthoracic echocardiography
VHD: valvular heart disease

## Data Availability

The datasets generated during and/or analyzed during this study are not publicly available due to the regulations from our Institutional Review Board but are available from the corresponding author on reasonable request.
