# Supplementary material for: Effects of a Cloud-Based Synchronous Telehealth Program on Valvular Regurgitation Regression: Retrospective Study
Source: J Med Internet Res. 2025 Apr 23;27:e68929. doi: 10.2196/68929 (PMC12059497; doi:10.2196/68929)
Supplement: Multimedia Appendix 1 [file jmir_v27i1e68929_app1.docx]

**Figure S1.** Flowchart of the mitral and tricuspid regurgitation cohort.

**
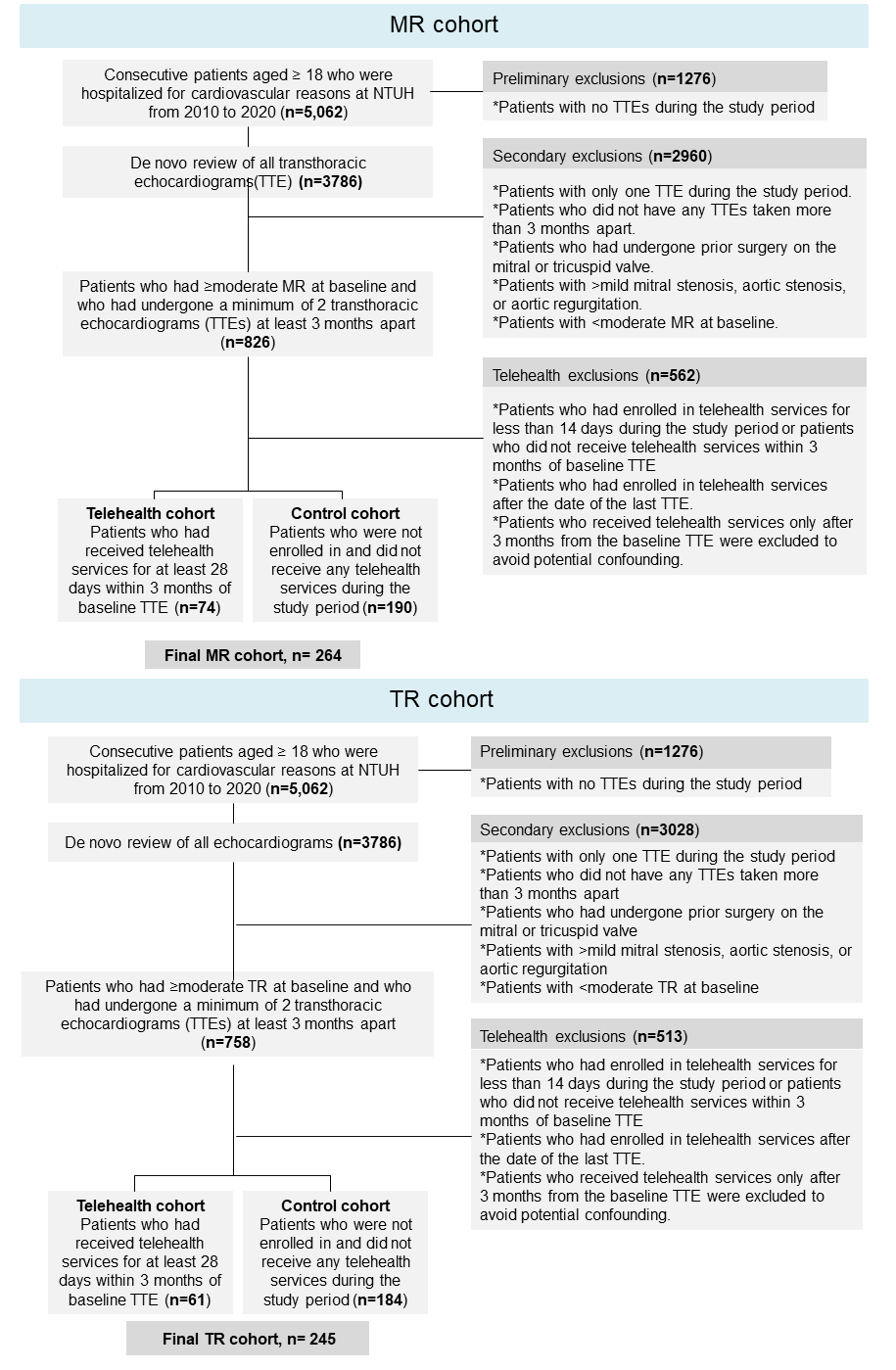
**

**Figure S2.** Time dependent changes in tricuspid regurgitation peak pressure gradient (TRPG). A significant time-dependent increase in TRPG was observed in the non-telehealth group (+0.03 mmHg per month, *P* =.01), while a significant interaction between telemedicine and follow-up duration (-0.09 mmHg per month, *P* < .001) suggests that the telehealth group experienced a modest but significant monthly TRPG decrease (-**
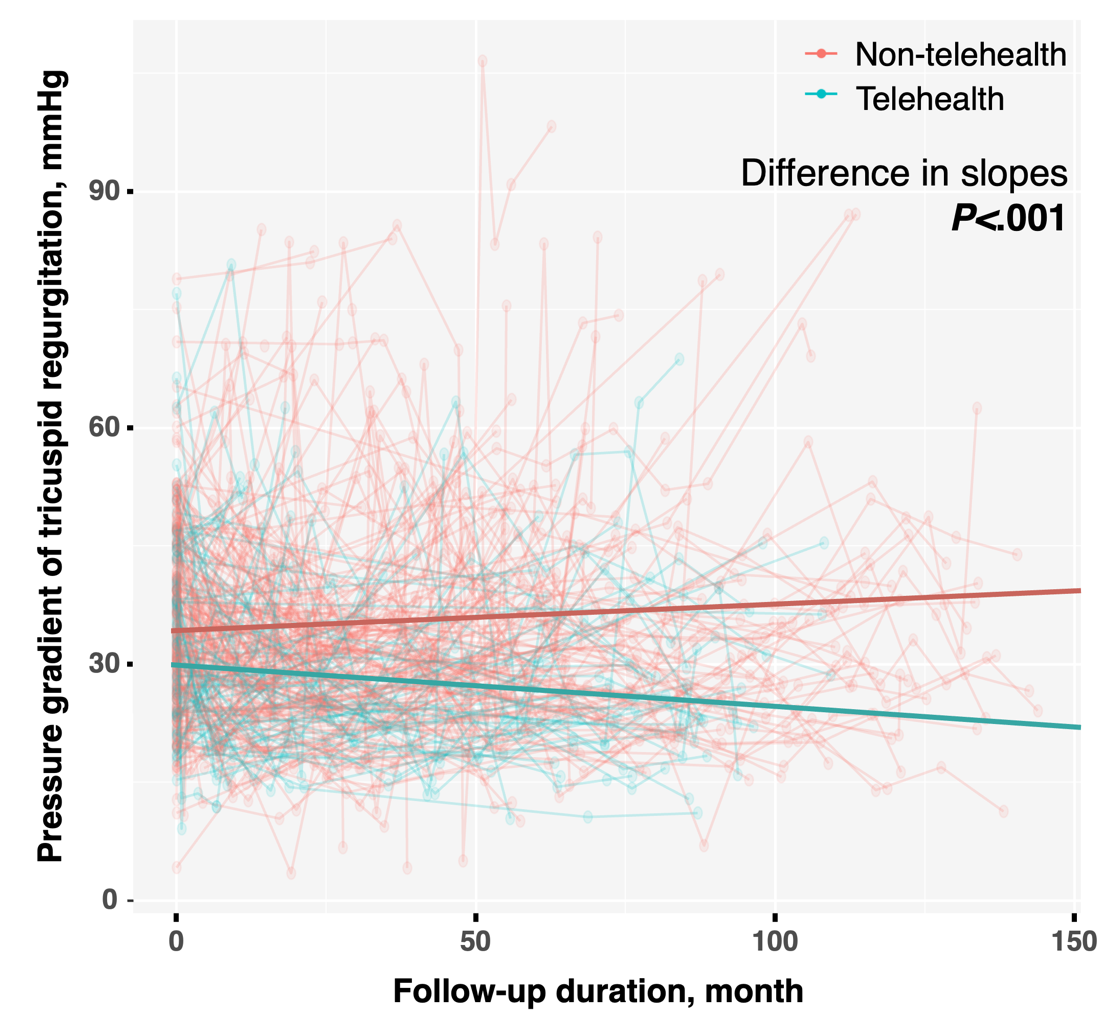
**0.05 mmHg per month).

**Table S1.** The comparison of clinical care received between the telehealth and non-telehealth groups.

| **Managements** | **Telehealth** | **Non-telehealth** |
| --- | --- | --- |
| Home-based biodata monitoring: manometer, oximeter, glucometer, single-lead ECG | V | X |
| Synchronous, and 24/7 internet-based data transmission platform | V | X |
| Automated ECG interpretation | V | X |
| Prompt detection of abnormal biodata values | V | X |
| Routine and on-demand telephone consultations by case managers* | V | X |
| Case managers promptly contact on-call cardiologists to discuss patient conditions and management plans | V | X |
| Timely dose adjustment of guideline-directed medical therapy | V | X |
| Routine outpatient follow-up | V | V |

ECG: electrocardiogram

*When abnormal biodata values were detected, case managers immediately contacted the patients to assess their condition. Otherwise, routine telephone follow-ups were conducted every 2–3 days. These follow-ups covered topics such as medication adherence (“Are you taking your medication regularly?”, “Have you experienced any side effects or issues with the medication?”), technical support for telehealth services (“Are there any problems with the App or monitoring devices?”), and potential dose adjustments, which were determined by telehealth center physicians based on ongoing biodata trends.

**Table S2.** Nurse level classification in Taiwan, according to the guidelines for the planning of clinical professional competence for primary care nursing personnel, Taiwan nurses Association.

Approximately 82% of nurse case managers in our center have achieved a level 2 or higher out of the available 4 levels, with an average job tenure of 8.9 years. In Taiwan, nurses of level 2 or above have completed two years of clinical work and successfully passed the N2 evaluation. They are qualified to participate in the care of critically ill patients. They participate in in-service education focusing on managing common medical conditions such as chest pain, abdominal pain, hypertension, and more before working in our center. Nurse case managers communicate with patients daily and make nursing notes after finishing the telecommunication. These nursing notes has been categorized into 6 predefined groups, including vital signs, clinical conditions, operation process, self-care, patient visit and equipment.

| **Nurse level** |  |
| --- | --- |
| N | Nurses with less than one year of clinical work experience. |
| N1 | Nurses with one year of clinical work experience, having completed N1 clinical professional competence training and passed the evaluation, capable of executing basic patient care. |
| N2 | Nurses with two years or more of clinical work experience, having completed N2 clinical professional competence training and passed the evaluation, able to participate in the care of critical patients.  Note: Critical patients are those in critical condition (requiring close observation and care) or with high dependency (categorized by levels A-I, A-II, B-I, B-II based on nursing dependency). |
| N3 | Nurses with three years or more of clinical work experience, having completed N3 clinical professional competence training and passed the evaluation, capable of providing comprehensive care for critical patients, problem analysis, and solving through case reports, as well as possessing teaching and assisting abilities for unit quality improvement. |
| N4 | Nurses with four years or more of clinical work experience, having completed N4 clinical professional competence training and passed the evaluation, capable of delivering comprehensive care for critical patients, along with teaching, participating in administrative tasks, and executing unit quality improvement. |

**Table S3. Baseline characteristics between patients with and without beta blocker use**

|  | **Beta blocker** | |  |
| --- | --- | --- | --- |
|  | **Yes** | **No** | *P* |
| Age | 69±13 | 66±14 | *.04* |
| Male | 76(62) | 46(37) | .10 |
| SBP, mmHg | 132±21 | 126±19 | *.02* |
| DBP, mmHg | 75±13 | 74±12 | .49 |
| AFib at TTE | 47(58) | 34(42) | .84 |
| CCI | 1.08±1.11 | 1.13±1.83 | .81 |
| Hypertension | 50(68) | 23(31) | *.01* |
| Diabetes mellitus | 32(58) | 23(42) | .85 |
| MI | 10(62) | 6(37) | .79 |
| Heart failure | 41(62) | 25(37) | .33 |
| Malignancy | 9(60) | 6(40) | 1.0 |
| Statin | 44(62) | 26(37) | .25 |
| Antiplatelet | 87(64) | 47(35) | *.006* |
| Anticoagulant | 39(62) | 23(37) | .28 |
| ACEi and ARB | 96(62) | 58(37) | *.03* |
| Beta blocker | — | — | — |
| CCB | 51(56) | 39(43) | .90 |
| Diuretics | 80(58) | 56(41) | .55 |
| LVEF, % | 57±17 | 61±13 | *.03* |
| LA dimension, cm | 4.5±0.8 | 4.3±0.9 | *.04* |
| LVEDD, mm | 50±8 | 48±9 | .34 |
| LVESD, mm | 34±11 | 32±9 | .10 |
| Baseline TR |  |  | .59 |
| Moderate | 117(83) | 85(80) |  |
| ≥ Moderate-severe | 23(16) | 20(19) |  |
| Baseline MR |  |  | .65 |
| None | 2(1) | 5(4) |  |
| Trivial | 2(1) | 2(1) |  |
| Mild | 36(25) | 33(31) |  |
| Mild-moderate | 20(14) | 12(11) |  |
| Moderate | 54(38) | 35(33) |  |
| Moderate-severe | 23(16) | 15(14) |  |
| Severe | 3(2) | 2(2) |  |
| **Last echocardiographic parameters** | | | |
| LVEF, % | 61±14 | 61±15 | .87 |
| LA dimension, cm | 4.5±0.9 | 4.4±1.1 | .28 |
| LVEDD, mm | 49±8 | 49±9 | .98 |
| LVESD, mm | 32±9 | 32±10 | .98 |
| MR at last follow-up ≥ moderate | 63(45) | 47(44) | .97 |

See Table 1 for abbreviations.

**Table S4.** Multivariable analysis for determinants of MR regression to less than moderate (N=97). Model 1 was presented in Table 1. Model 2, which excluded “telehealth”, shows that the use of beta-blocker was marginally associated with MR regression.

|  | **Model 1: Original model** | | **Model 2** | |
| --- | --- | --- | --- | --- |
|  | HR (95% CI) | HR (95% CI) |  |  |
| Telehealth vs. non-telehealth | 2.20(1.35-3.58) | *.001* | *─* | *─* |
| Age, year | 0.99(0.98-1.01) | .94 | 0.99(0.98-1.01) | .73 |
| Male | 1.08(0.69-1.68) | .72 | 1.04(0.67-1.62) | .84 |
| Beta blocker | 1.32(0.81-2.15) | .26 | 1.60(0.99-2.57) | .05 |
| CCB |  |  |  |  |
| Baseline LA dimension, cm | 0.78(0.57-1.07) | .13 | 0.72(0.52-0.99) | *.04* |
| Baseline LVEF, % | 0.99(0.97-1.00) | .25 | 0.98(0.97-1.00) | .07 |
| TRPG |  |  |  |  |
| Baseline MR severity (Ref: moderate) |  |  |  |  |
| Moderate-severe | 0.63(0.31-1.25) | .18 | 0.60(0.30-1.19) | .15 |
| Severe | 0.40(0.05-3.25) | .39 | 0.42(0.05-3.33) | .41 |
| Primary MR vs FMR | 1.34(0.67-2.68) | .40 | 1.19(0.60-2.37) | .60 |
| Baseline TR< moderate | 1.28(0.83-2.00) | .25 | 1.16(0.75-1.80) | .48 |
| Time-dependent PCI | 1.23(0.76-1.99) | .38 | 1.42(0.89-2.28) | .13 |

See Table 1 for abbreviations.

**Table S5.** Multivariable determinants for regression of mitral regurgitation(MR) to less than moderate in patients with baseline moderate MR only (N=200, 84 events).

|  | HR (95% CI) | *P* |
| --- | --- | --- |
| Telehealth vs. non-telehealth | 2.56(1.56-4.21) | *<.001* |
| Age, year | 0.99(0.98-1.01) | .74 |
| Male | 1.17(0.73-1.89) | .49 |
| Baseline LA dimension, cm | 0.83(0.59-1.16) | .28 |
| Baseline LVEF, % | 0.99(0.98-1.01) | .91 |
| Time dependent PCI | 1.36(0.83-2.21) | .21 |
| Baseline TR less than moderate | 1.53(0.95-2.46) | .07 |

See Table 1 for abbreviations.

**Table S6.** Univariable and multivariable determinants for mitral regurgitation regression to less than moderate, in patients admitted before COVID outbreak (N=98).

|  | **Univariable analysis** | | **Multivariable analysis** | |
| --- | --- | --- | --- | --- |
|  | HR (95% CI) | *P* | HR (95% CI) | *P* |
| **Telehealth vs. non-telehealth** | 2.90 (1.87-4.48) | *<.001* | 2.32(1.43-3.75) | *<.001* |
| Age, year | 0.99 (1.00-1.00) | .21 | 0.99(0.97-1.01) | .43 |
| Male | 1.18 (0.78-1.77) | .41 | 1.11(0.71-1.73) | .62 |
| SBP, mmHg | 1.01 (0.98-1.04) | .43 |  |  |
| DBP, mmHg | 1.03 (0.98-1.08) | .12 |  |  |
| CCI | 1.07 (0.94-1.22) | .27 |  |  |
| AFib at TTE | 1.37 (0.82-2.30) | .20 |  |  |
| ACEi and ARB | 0.96 (0.63-1.47) | .87 |  |  |
| Diuretics | 1.22 (0.81-1.84) | .32 |  |  |
| Statin | 1.14 (0.74-1.74) | .53 |  |  |
| Antiplatelets | 1.18 (0.77-1.79) | .42 |  |  |
| Beta blocker | 1.57 (1.01-2.44) | *.03* | 1.38 (0.84-2.24) | .19 |
| CCB | 1.09 (0.72-1.65) | .66 |  |  |
| Baseline LA dimension, cm | 0.74 (0.56-0.98) | *.03* | 0.77 (0.56-1.06) | .11 |
| Baseline LVEF, % | 0.98 (0.97-0.99) | *.02* | 0.99 (0.97-1.00) | .15 |
| Baseline LVEDD, mm | 0.99 (0.96-1.01) | .61 |  |  |
| Baseline LVESD, mm | 1.01 (0.99-1.02) | .25 |  |  |
| TRPG | 1.00 (0.98-1.02) | .57 |  |  |
| Baseline MR severity (Ref: moderate) |  |  |  |  |
| Moderate-severe | 0.51 (0.27-0.93) | *.03* | 0.61 (0.30-1.22) | .16 |
| Severe | 0.23 (0.03-1.78) | .16 | 0.42 (0.05-3.35) | .41 |
| Primary MR vs FMR | 0.75 (0.40-1.41) | .36 | 1.29(0.64-2.60) | .45 |
| Baseline TR< moderate | 1.44(0.96-2.17) | .07 | 1.15(0.74-1.78) | .52 |
| Time-dependent PCI | 1.47 (0.95-2.28) | .09 | 1.27 (0.79-2.06) | .31 |

See Table 1 for abbreviations.

**Table S7.** Baseline characteristics of TR regressors (< moderate) vs. non-regressors (≥ moderate), and telehealth vs. non-telehealth groups (N= 245).

|  | **Regressors**  **(N=87)** | **Non-regressors**  **(N=158)** | *P* | **Telehealth (N=61)** | **non-telehealth (N=184)** | *P* |
| --- | --- | --- | --- | --- | --- | --- |
| Age | 65±16 | 70±12 | *.01* | 64±15 | 70±13 | *.008* |
| Male | 46(52) | 76(48) | .47 | 33(54) | 89(48) | .43 |
| SBP, mmHg | 130±22 | 130±20 | .85 | 128±22 | 130±20 | .39 |
| DBP, mmHg | 76±15 | 75±12 | .46 | 78±14 | 75±12 | .13 |
| Telehealth | 28(32) | 33(20) | *.05* | **—** | **—** | **—** |
| AFib at TTE | 11(12) | 32(20) | .12 | 17(28) | 64(35) | .31 |
| CCI | 1(0-2) | 1(0-2) | .62 | 1(0-2) | 1(0-2) | .85 |
| Hypertension |  |  |  | 21(34) | 52(28) | .36 |
| Diabetes mellitus | 25(29) | 30(19) | .08 | 12(20) | 43(23) | .54 |
| MI | 6(7) | 10(6) | .86 | 4(7) | 12(7) | 1.0* |
| Heart failure | 21(24) | 45(28) | .46 | 21(34) | 45(24) | .13 |
| Malignancy | 6(7) | 9(6) | .70 | 5(8) | 10(5) | .45 |
| Statin | 27(31) | 43(27) | .52 | 18(29) | 52(28) | .85 |
| Antiplatelet | 53(60) | 81(51) | .14 | 34(55) | 100(51) | .85 |
| Anticoagulant | 20(22) | 42(26) | .53 | 20(32) | 42(22) | .12 |
| ACEi and ARB | 56(64) | 98(62) | .71 | 38(62) | 116(63) | .91 |
| Beta blocker | 56(64) | 84(53) | .08 | 38(62) | 102(55) | .34 |
| CCB | 26(29) | 64(40) | .09 | 20(32) | 70(38) | .46 |
| Diuretics | 43(49) | 93(58) | .15 | 33(54) | 103(55) | .79 |
| **Baseline echocardiographic parameters** | | | |  |  |  |
| LVEF, % | 58±16 | 59±15 | .49 | 55±18 | 61±15 | *.02* |
| LA dimension, cm | 4.1±0.7 | 4.5±0.9 | *<.001* | 4.1±0.8 | 4.5±0.9 | *.007* |
| LVEDD, mm | 49±9 | 50±9 | .62 | 50±10 | 50±9 | .96 |
| LVESD, mm | 34±11 | 34±11 | .92 | 36±13 | 33±10 | .21 |
| Baseline TR |  |  | *<.001* |  |  | .90 |
| Moderate | 82(94) | 120(75) |  | 51(84) | 151(82) |  |
| Moderate-severe | 5(5) | 31(19) |  | 8(13) | 28(15) |  |
| Severe | 0(0) | 7(4) |  | 2(3) | 5(3) |  |
| Baseline MR |  |  | .81 |  |  | .09 |
| None | 3(3) | 4(2) |  | 1(2) | 6(3) |  |
| Trivial | 2(2) | 2(1) |  | 2(3) | 2(1) |  |
| Mild | 24(27) | 45(28) |  | 14(23) | 55(30) |  |
| Mild-moderate | 15(17) | 17(10) |  | 11(18) | 21(11) |  |
| Moderate | 28(32) | 61(38) |  | 28(46) | 61(33) |  |
| Moderate-severe | 13(14) | 25(15) |  | 5(8) | 33(18) |  |
| Severe | 2(2) | 3(1) |  | 0(0) | 5(3) |  |
| MR at last follow-up ≥moderate | 26(29) | 84(53) | *<.001* | 22(36) | 88(48) | .10 |
| **Last echocardiographic parameters** | | | |  |  |  |
| LVEF, % | 64±13 | 60±15 | *.05* | 62±14 | 61±15 | .86 |
| LA dimension, cm | 4.0±0.9 | 4.7±1.1 | *<.001* | 4.1±0.8 | 4.6±1.1 | *<.001* |
| LVEDD, mm | 48±8 | 50±10 | .07 | 50±9 | 49±9 | .65 |
| LVESD, mm | 31±9 | 34±11 | *.04* | 33±11 | 33±10 | .74 |
| MR at last follow-up ≥ moderate | 26(30) | 84(53) | *<.001* | 22(36) | 88(48) | .10 |
| PCI after baseline | 27(31) | 20(13) | *<.001* |  |  |  |

See Table 1 for abbreviations.

**Table S8.** Univariable and multivariable determinants for tricuspid regurgitation regression to < moderate (N=87).

|  | **Univariable analysis** | | **Multivariable analysis** | |
| --- | --- | --- | --- | --- |
|  | HR (95% CI) | *P* | HR (95% CI) | *P* |
| Age, year | 0.99(0.97-1.00) | .24 | 0.99(0.97-1.01) | .49 |
| Male | 0.92(0.59-1.41) | .70 | 0.82(0.49-1.36) | .44 |
| SBP, mmHg | 1.00(0.99-1.01) | .20 |  |  |
| DBP, mmHg | 1.00(0.98-1.02) | .73 |  |  |
| Telehealth vs. non-telehealth | 1.57(0.99-2.50) | *.05* | 1.28(0.76-2.16) | .33 |
| CCI | 1.08(0.93-1.22) | .26 |  |  |
| AFib at TTE | 0.62(0.33-1.18) | .15 |  |  |
| ACEi and ARB | 1.16(0.74-1.81) | .50 |  |  |
| Diuretics | 1.06(0.69-1.64) | .76 |  |  |
| Statin | 1.19(0.75-1.88) | .45 |  |  |
| Antiplatelets | 1.33(0.86-2.06) | .19 |  |  |
| Beta blocker | 1.61(1.02-2.53) | *.03* | 1.64(1.00-2.68) | *.048* |
| CCB | 0.91(0.57-1.45) | .70 |  |  |
| LA dimension, cm | 0.74(0.56-0.98) | *.03* | 0.81(0.58-1.12) | .22 |
| LVEF, % | 0.98(0.97-1.00) | .08 |  |  |
| LVEDD, mm | 0.99(0.97-1.02) | .78 |  |  |
| LVESD, mm | 1.00(0.98-1.02) | .46 |  |  |
| TRPG | 1.00(0.98-1.01) | .81 |  |  |
| **Baseline TR severity (moderate TR as reference)^a^** |  |  |  |  |
| ≥Moderate-severe | 0.28(0.11-0.70) | *.006* | 0.28(0.10-0.78) | *.01* |
| Baseline MR < moderate | 1.18(0.77-1.81) | .43 |  |  |
| Time-dependent PCI | 1.82(1.15-2.90) | *.01* | 1.47(0.87-2.49) | .14 |

See Table 1 for abbreviations.

^a^There was only 7 patients having severe TR at baseline thus we categorized patients into moderate or ≥Moderate-severe TR

**Table S9.** Univariable and multivariable determinants for all-cause death in tricuspid regurgitation cohort (113 events).

|  | **Univariable analysis** | | **Multivariable analysis** | |
| --- | --- | --- | --- | --- |
|  | HR (95% CI) | *P* | HR (95% CI) | *P* |
| **Regressors vs non-regressors** | 0.69(0.46-1.03) | .06 |  |  |
| Age, year | 1.05(1.03-1.07) | *<.001* | 1.04(1.02-1.07) | *<.001* |
| Male | 0.91(0.63-1.32) | .63 |  |  |
| SBP, mmHg | 1.01(1.00-1.02) | *.03* | 1.00(0.99-1.01) | .25 |
| DBP, mmHg | 0.99(0.97-1.01) | .25 |  |  |
| **Telehealth vs. non-telehealth** | 0.50(0.29-0.84) | *.005* | 0.63(0.35-1.13) | .11 |
| CCI | 1.08(0.96-1.19) | .17 |  |  |
| AFib at TTE | 1.48(1.01-2.16) | *.04* | 1.13(0.72-1.77) | .58 |
| ACEi and ARB | 1.63(1.08-2.45) | *.01* | 1.16(0.73-1.82) | .51 |
| Diuretics | 2.31(1.54-3.46) | *<.001* | 1.49(0.92-2.42) | .09 |
| Statin | 0.89(0.59-1.35) | .61 |  |  |
| Antiplatelets | 1.05(0.73-1.53) | .76 |  |  |
| Beta blocker | 1.42(0.97-2.08) | .06 |  |  |
| CCB | 1.67(1.15-2.42) | *.006* | 1.18(0.77-1.81) | .43 |
| Baseline LA dimension, cm | 1.43(1.17-1.74) | *<.001* | 1.23(0.95-1.58) | .10 |
| Baseline LVEF, % | 0.98(0.97-0.99) | *.01* | 0.97(0.96-0.99) | *.004* |
| Baseline LVEDD, mm | 1.01(0.99-1.03) | .07 |  |  |
| Baseline LVESD, mm | 1.01(1.00-1.03) | *.03* |  |  |
| TRPG | 1.01(0.99-1.02) | .12 |  |  |
| Baseline MR ≥Moderate | 1.38(0.94-2.02) | .08 |  |  |
| Baseline TR ≥ moderate-severe^a^ | 0.96(0.58-1.57) | .88 |  |  |
| Time-dependent PCI | 0.91(0.56-1.48) | .70 |  |  |

See Table 1 for abbreviations.

^a^There was only 7 patients having severe TR at baseline thus we categorized patients into moderate or ≥Moderate-severe TR

The Pearson correlation coefficient was -0.65 between LVEF and LVEDD, and was -0.89 between LVEF and LVESD(both *P*<.001).
